# Expression of HIWI in human esophageal squamous cell carcinoma is significantly associated with poorer prognosis

**DOI:** 10.1186/1471-2407-9-426

**Published:** 2009-12-08

**Authors:** Wei He, Zhihui Wang, Qi Wang, Qingxia Fan, Chengcao Shou, Junsheng Wang, Karl-Erik Giercksky, Jahn M Nesland, Zhenhe Suo

**Affiliations:** 1 Department of Oncology, the First Affiliated Hospital of Zhengzhou University, Zhengzhou, 450052, PR China; 2 Divisions of Pathology, The Norwegian Radium Hospital, Oslo University Hospital, Montebello, Oslo, 0310, Norway; 3 Faculty Division The Norwegian Radium Hospital, Faculty of Medicine, University of Oslo, Oslo, 0316, Norway; 4 Department of Respiratory Medicine, the Second Hospital Affiliated to Dalian Medical University, Dalian, 116023, PR China; 5 Department of Biochemistry and Molecular Biology, Peking University School of Oncology, Beijing Institute for Cancer Research, Beijing, 100142, PR China; 6 Department of Oncology, Anyang Tumor Hospital, Anyang, 455000, PR China; 7 Department of Surgery, The Norwegian Radium Hospital, Oslo University Hospital, Montebello, Oslo, 0310, Norway; 8 Faculty Division The Norwegian Radium Hospital, Faculty of Medicine, University of Oslo, Oslo, 0316, Norway

## Abstract

**Background:**

HIWI, the human homologue of Piwi family, is present in CD34^+ ^hematopoietic stem cells and germ cells, but not in well-differentiated cell populations, indicating that HIWI may play an impotent role in determining or maintaining stemness of these cells. That HIWI expression has been detected in several type tumours may suggest its association with clinical outcome in cancer patients.

**Methods:**

With the methods of real-time PCR, western blot, immunocytochemistry and immunohistochemistry, the expression of HIWI in three esophageal squamous cancer cell lines KYSE70, KYSE140 and KYSE450 has been characterized. Then, we investigated HIWI expression in a series of 153 esophageal squamous cell carcinomas using immunohistochemistry and explored its association with clinicopathological features.

**Results:**

The expression of HIWI was observed in tumour cell nuclei or/and cytoplasm in 137 (89.5%) cases, 16 (10.5%) cases were negative in both nuclei and cytoplasm. 86 (56.2%) were strongly positive in cytoplasm, while 49 (32.0%) were strongly positive in nuclei. The expression level of HIWI in cytoplasm of esophageal cancer cells was significantly associated with histological grade (*P *= 0.011), T stage (*P *= 0.035), and clinic outcome (*P *< 0.001), while there was no correlation between the nuclear HIWI expression and clinicopathological features.

**Conclusion:**

The expression of HIWI in the cytoplasm of esophageal cancer cells is significantly associated with higher histological grade, clinical stage and poorer clinical outcome, indicating its possible involvement in cancer development.

## Background

Cancer therapeutics has experienced remarkable improvement, but still, cancer is a leading cause of death worldwide. During the past 7 years, cells with stem cell-like features so-called cancer stem cells have been identified in acute myeloic leukemia, breast cancer, melanoma and tumors of central nervous system, etc[1,2], which support the concept that cancer stem cells are essential to the development and perpetuation of human cancer [3-5]. It has been suggested that deregulation of stem cell self-renewal may cause development of malignancies because cancer cells and stem cells share several similarities, such as the ability of self-renewal without loss of proliferation capacity, relative resistance to drugs, radiation, and apoptosis. In addition, cancer stem cells have been shown to express typical markers of normal stem cells [2]. These similarities suggest the expression of genes determining cell renewal and stemness in cancer cells.

HIWI, also called PIWI1, is a human homologue of the PIWI family. The PIWI family, a subfamily of Argonaute proteins, have so far been described in jellyfish, *Caenorhabditis elegans*, zebrafish, drosophila, mice as well as many other species [6-12]. The PIWI family are highly conserved during evolution and play an important role in stem cell self-renewal, division, gametogenesis, germ cell proliferation, RNA silencing and translational regulation in diverse organisms [13-17]. The impressive biological capacities of PIWI family suggest that they may be key elements in the regulation of stem cells and germ cells.

As a human member of PIWI family, HIWI is present in CD34^+ ^hematopoietic stem cells but not in well-differentiated cell populations, indicating that HIWI may be a candidate protein in determination or regulation of HSC development[18]. Qiao *et al*[19] found that HIWI was also expressed in spermatocytes and round spermatids during spermatogenesis and increased in testicular seminoma, suggesting that deregulation of HIWI expression may contribute to the occurrence and development of tumour. Furthermore, HIWI expression has been reported in different solid tumours such as gastric carcinoma, soft-tissue sarcoma (STS) and pancreas adenocarcinoma [16,19-21]. Thus, HIWI may be present in other types of cancer cells and involved in the development of various cancer forms.

Esophageal squamous cell carcinoma (ESCC) is one of the leading causes of cancer-related death throughout the world and the incidence of which ranks fourth as cause of death in China and seventh in the world[22]. Although surgery is potential curative for early stage esophageal cancer, it is still a limited clinical option for most patients presenting with advanced disease. Analysis of the stem cell-associated gene in this malignancy may provide important clues to the understanding of this disease development.

In the present study, we investigated the expression of HIWI in a cohort of 153 patients with ESCC and explored its clinicopathological and survival associations. We did find that the expression of HIWI was significantly associated with higher T stage, higher histological grade and poorer clinical survival, indicating that this protein may exert positively influence on tumour cell stemness, which in turn results in negative clinical course for ESCC patients.

## Methods

### Patients and Materials

In this retrospective study which was approved by the Ethics Committee of Anyang Tumour Hospital and Anyang Hygiene Bureau, we randomly selected 153 consecutive ESCC patients who underwent potentially curative surgery without preoperative chemotherapy or radiotherapy during the period of 1989-1994 at The Anyang Tumour Hospital, Henan, China. Among them, 93 are men and 60 are women, ranging from 33-73 years of age with a mean age of 56.4 years. According to International Union against Cancer (UICC) 2003 standard, 100 cases were classified as stage I or II and 53 cases as stage III or IV. The patients' information and tumour parameters are listed in Table 1. All the patients were followed at the Anyang Tumour Hospital until May 2004 supported by an international collaboration project between Anyang Tumour Hospital and The Norwegian Radium Hospital[23]. A total of 97 (63.4%) patients died during the follow-up period. In addition, 10 "non-cancerous" surrounding esophageal epithelium specimens were also applied for the HIWI immunostaining. Surgically removed specimens were routinely fixed in buffered formalin and embedded in paraffin block for clinical diagnosis and reclassification for this study.

**Table 1:** Clinicopathological features and HIWI expression in esophageal squamous cell carcinoma

Parameters	Cases (%)	Cytoplasma			Nucleus				Cytoplasma and Nucleus		
		
		Low	High	*P*	Low	High	*P*	Low	Moderate	High	*P*
Age				0.683			0.830				0.727
<51	48 (31.4)	21	27		31	17		13	26	9	
51-60	52 (34.0)	25	27		36	16		19	23	10	
>60	53 (34.6)	21	32		37	16		14	30	9	
Gender				0.448			0.666				0.251
Male	93 (60.8)	43	50		62	31		31	43	19	
Female	60 (39.2)	24	36		42	18		15	36	9	
Location				0.813			0.311				0.556
Upper	14 (9.2)	5	9		7	7		3	6	5	
Middle	101 (66.0)	44	57		71	30		31	53	17	
Lower	35 (22.9)	16	19		24	11		11	18	6	
Missing	3 (2.0)	2	1		2	1		1	2	0	
Size				0.778			0.881				0.705
<31 mm	25 (16.3)	11	14		18	7		7	15	3	
31-60 mm	105 (68.6)	48	57		73	32		34	53	18	
>60 mm	14 (9.2)	5	9		9	5		4	6	4	
Missing	9 (5.9)	3	6		4	5		1	5	3	
Lymph node metastasis				0.904			0.232				0.593
-	99 (64.7)	43	56		64	35		27	53	19	
+	54 (35.3)	24	30		40	14		19	26	9	
UICC stage				0.074			0.148				0.662
I	0	0	0		0	0		0	0	0	
II	100 (65.4)	49	51		64	36		32	49	19	
III	53 (34.6)	18	35		40	13		14	30	9	
IV	0	0	0		0	0		0	0	0	
T stage				0.035*			0.278				0.002*
I	5 (3.3)	4	1		4	1		3	2	0	
II	39 (25.5)	23	16		31	8		19	16	4	
III	100 (65.4)	37	63		63	37		24	52	24	
IV	9 (5.9)	3	6		6	3		0	9		
Histological grade				0.011*			0.352				0.126
Well	53 (34.6)	31	22		37	16		21	26	6	
Moderate	60 (39.2)	25	35		37	23		17	28	15	
Poor	40 (26.1)	11	29		30	10		8	25	7	
Patients at follow-up				<0.001*			0.981				0.001*
Alive	56 (36.6)	35	21		38	18		27	19	10	
Dead	97 (63.4)	32	65		66	31		19	60	18	

* *P *< 0.05

The human ESCC cell lines KYSE70, KYSE140 and KYSE450 (DSMZ, Germany) were cultured in RPMI 1640 medium supplemented with 10% fetal bovine serum at 37°C under 5% CO_2 _and saturated moisture. The sample cells were collected and counted by trypan blue exclusion using a hemocytometer.

### Immunocytochemistry

Immunocytochemistry was performed on these three cell lines. Simply, the cultured cells were detached and the cell suspension was centrifuged at 2000 rpm for 10 min. The supernatant was removed before the sediment was mixed with 2-3 drops plasma and 2 drops thrombin for 1 min. 4% buffered formalin was then added to the coagulated cell mass, which was then fixed for 30 min and placed in linen paper for further conventional paraffin cytoblock preparation. 4 μm sections were cut from the paraffin-embedded cytoblocks and immunostained according to the same procedure as tissue blocks described below.

### Tissue Arrays

Multitissue array blocks were made with the MTA-1 manual tissue arrayer (Beecher Instruments Inc., Sun Prairie, WI, USA). Simply, 4 μm sections from the routinely made paraffin blocks were stained with H&E and re-evaluated to confirm the diagnosis and to identify three representative tumour areas. Then, the related paraffin blocks were subsequently oriented and marked. From these blocks, tissue cores with a diameter of 0.6 mm were punched and arrayed in triplicate on a recipient paraffin block. After the block construction was completed, the block was placed into a 40°C oven overnight for tightening the cylinders by slightly melting paraffin. 4 μm sections of these tissue array blocks were cut and placed on charged Super-Frost Plus glass slides, and dried at 60°C oven for 2-4 hours. These sections were used for immunohistochemical analysis. For those samples their tissue array materials were not representative or not available, the paraffin-embedded conventional sections were used for additional immunohistochemistry analyses as well.

### Immunohistochemistry

Immunohistochemical analysis for HIWI was performed on 4 μm sections. The Envision Plus detection system (Dako, Carpinteria, CA, USA) was used for the detection of immunostaining. The sections were deparaffinized in xylene and then microwaved in 10 mM citrate buffer (pH 6.0) to unmask the epitopes. Endogenous peroxidase activity was blocked by incubation with 0.03% hydrogen peroxide in methanol for 5 min. Sections were incubated with a monoclonal mouse anti-HIWI antibody (developed at the Professor Chengchao Shou's lab) for 30 min at room temperature. The specificity for the HIWI antibody was previously reported and the working condition applied in this study was the same as previously published[16]. After gently rinsed three times with washing buffer, the sections were incubated with the peroxidase-labeled polymer conjugated to goat anti-mouse IgG (Dako, Carpinteria, CA, USA) for 30 min before stained for 5 min with 3'3-diaminobenzidine tetrahydrochloride (DAB), counterstained by hematoxylin, dehydrated, and mounted in Diatex. A known HIWI positive seminoma was used as positive control while the same concentration of non-immune mouse IgG was applied as negative control. All controls gave satisfactory results.

### HIWI immunostaining scoring

Tumor cell immunoreactivity was scored according to both nuclear and cytoplasmic staining. Both the extent of staining (percentage of positive cells) and the intensity of the reaction were taken into account. Briefly, the extent of positivity was scored as 0 when no positive cell was observed; 1 when the percentage of positive cells was <10%; 2 when it was 10-50%; and 3 when it was >50%. The intensity was scored as 0 when no positive cells identified; 1, weak; 2, moderate; and 3, strong staining. Multiplying the extent by intensity gave the following immunohistochemical staining grades as 0, 1, 2, 3, 4, 6 and 9. For statistical analyses, the grades 0, 1, 2 and 3 were considered as no or weakly stained and scored as 0, the grades 4, 6 and 9 were considered as strongly stained and scored as 1. Then all the specimens were attributed to three groups: "low", both nuclei and cytoplasm were scored as 0; "moderate", either nuclei or cytoplasm was scored as 1; "high", both nuclei and cytoplasm were scored as 1. The evaluation was performed independently by three experienced investigators who were unaware of the related clinical information and the conflicting scores were resolved at a discussion microscope.

### Western Blotting Analysis

25 mg of protein samples were separated on a 10% SDS-acrylamide gel (Bio-Rad) for 1 h at 150 V, and the proteins were transferred to a nitrocellulose membrane (Whatman, Kent, UK). After blocking in 5% fat-free milk, the membrane was treated with the dilution of the primary antibody overnight at 4°C and the dilution of the secondary IgG-horseradish peroxidase (HRP) conjugated antibody for 1 h at room temperature. The antibodies used for immunohistochemistry were applied for Western blotting. All the antibodies were diluted in phosphate buffered saline containing 5% Blotto and 0.1% Tween-20. The stained membranes were visualized by enhanced chemiluminescence reaction using the ECL Plus (GE Healthcare, Fairfild, CT, USA). Western blot experiments were repeated at least three times on every sample with similar results.

### Statistical Analysis

Bivariate association between ordinal variables was assessed using Spearman's correlation (exact version). For categorical data, Pearson's χ^2 ^test was used. All tests of statistical significance were two-sided. Overall survival was calculated from date of diagnosis to the date of death or May 1^st^, 2004. Survival curves were plotted according to the Kaplan-Meier method, and the log-rank test was used to determine significant differences among groups (pooled over strata for two groups; pairwise over strata for three groups). Multivariate analysis according to Cox's proportional hazards regression model adjusted clinicopathological factors (age, gender, tumour location, tumour size, lymph node metastasis, histological grade, and T stage) was performed to assess which tumor variables were independently correlated with overall survival. Statistical analyses were performed using the SPSS 16.0 package and *p *< 0.05 was considered as statistically significant.

## Results

### Characterization of HIWI in Esophageal cancer Cell Lines

**Figure 1:**
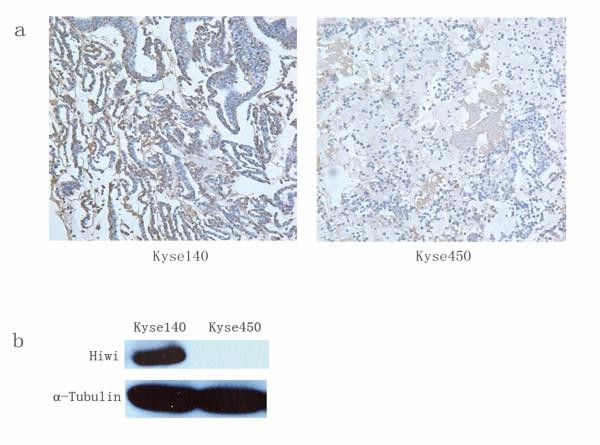
**a: Immunocytochemistry of HIWI in Kyse140 and Kyse450 cell lines; b: Western-blot analysis of HIWI in these two cell lines**. α-Tubulin was set as a loading control.

To examine the specificity and working concentration of anti-HIWI Mab, Western blotting and immunocytochemistry analysis were performed on two human esophageal squamous cell carcinoma cell lines Kyse140, Kyse450. Figure 1b shows typical 98 KD HIWI bands on Kyse140, which is in agreement with the NP_004755 HIWI characterization on NCBI. But there was no HIWI expression in Kyse450 cells. Immunocytochemistry of the cell lines cytoblock paraffin sections (Figure 1a) gave similar results as shown by Western blotting, indicating that HIWI expression varies in cancer cell lines.

### Pattern of HIWI Expression in Tumour samples

**Figure 2:**
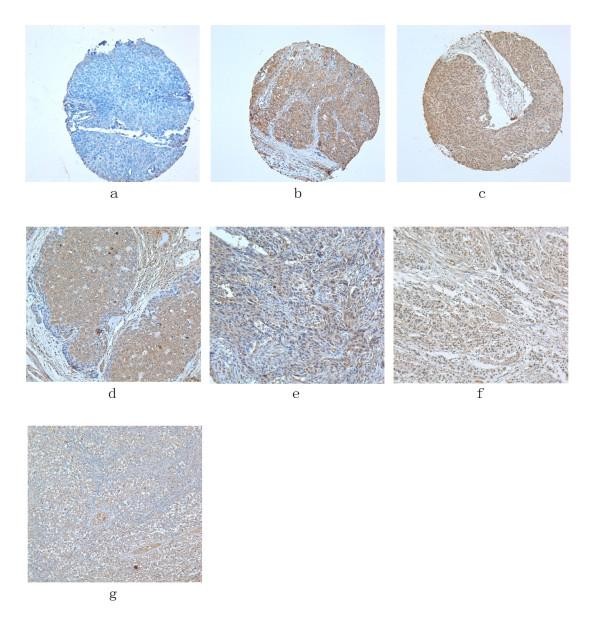
**Immunohistochemistry of HIWI in esophageal squamous cell carcinomas (×200)**. a-c: Tissue array sections; d-g: paraffin-embedded conventional sections. a: Negative; b: Strong cytoplasmic staining; c: Strong staining in both cytoplasm and nuclei; d: Strong cytoplasmic staining; e: Strong staining in both cytoplasm and nuclei; f: Strong nuclear staining; g: Strong cytoplasmic staining in seminoma (positive control).

HIWI immunoreactivity was observed in both nucleus and cytoplasm (Figure 2). Among the 153 specimens, 37 were negative for cytoplasmic staining and 30 were scored as 1, 2 or 3, which were classified as either negative or weakly positive for cytoplasmic immunostaining group (67 in total, 43.8%). 86 (56.2%) were scored as 4, 6 or 9 and classified as strongly positive group. For nuclear staining, 78 were negative and 26 were scored as 1, 2 or 3, giving a total of 104 (68.0%) specimens classified as either negative or weakly positive group. 49 (32.0%) were scored as 4, 6 or 9, and classified as strongly positive group (Table 2). Totally, 46 (30.1%) samples exhibited negative or weak HIWI expression in both cytoplasm and nuclei, and were classified as negative/weakly positive group; 58 cases (37.9%) were only strongly immunoreactive in cytoplasm, and 21 (13.7%) only strongly immunoreactive in nuclei, classified as moderate expression group together; 28 cases (18.3%) showed strong immunoreactivity in both cytoplasm and nuclei, classified as strongly positive group (Table 1). There was no positive expression in 10 "non-cancerous" surrounding esophageal epithelial sections.

**Table 2:** Immunohistochemical staining results for HIWI

Score	Cytoplasm		Nucleus	
	
	n	(%)	n	(%)
0	37	(24.2)	78	(51.0)
1	1	(0.7)	0	(0.0)
2	5	(3.3)	3	(2.0)
3	24	(15.7)	23	(15.0)
4	5	(3.3)	5	(3.3)
6	41	(26.8)	23	(15.0)
9	40	(26.1)	21	(13.7)

Total	153	(100.0)	153	(100.0)

### Relationship Between HIWI Expression and Clinicopathological Features

IHC was employed to investigate the association between HIWI expression and clinicopathological features in the 153 esophageal squamous cell carcinoma patients (Table 1). The expression level of HIWI in cytoplasm was significantly associated with histological grade (*P *= 0.011) and T stage (*P *= 0.035), but not associated with age, gender, tumour location, tumour size, lymph node metastasis and clinical stage, while there was no correlation between the expression level of HIWI in nucleus and the same clinicopathological features.

### Association between HIWI immunoreactivity and Survival

**Figure 3:**
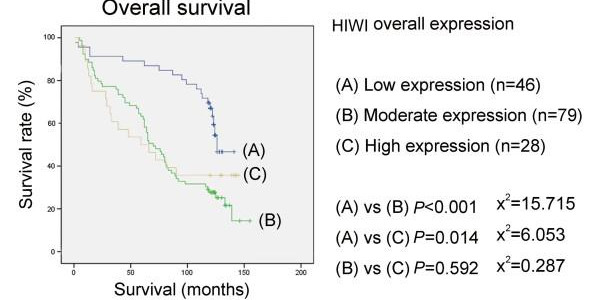
**Kaplan-Meier survival curves for esophageal squamous cell carcinoma patients with regard to HIWI protein expression: overall survival versus HIWI expression in both cytoplasm and nuclei**. Patients with low HIWI expression tumours showed a better overall survival than patients with moderate or high expression tumours, while there was no difference between the moderate expression group and high expression group. *p *values were determined by the log-rank test.

**Figure 4:**
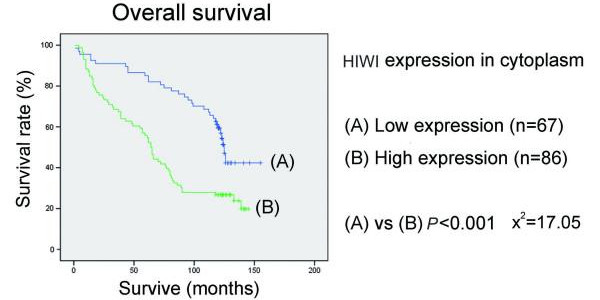
**Kaplan-Meier survival curves for esophageal squamous cell carcinoma patients with regard to HIWI protein expression: overall survival versus HIWI expression in cytoplasm**. Patients with high cytoplasmic HIWI expression tumours showed a poorer outcome than that with low cytoplasmic expression tumours. *p *values were determined by the log-rank test.

**Figure 5:**
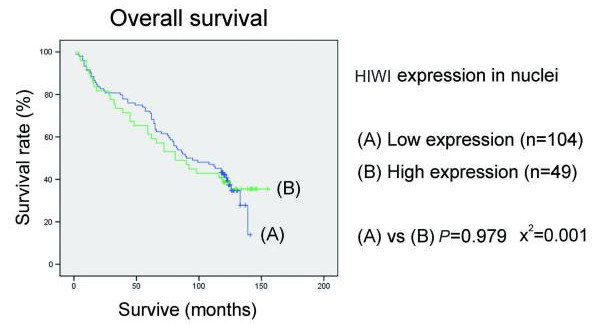
**Kaplan-Meier survival curves for esophageal squamous cell carcinoma patients with regard to HIWI protein expression: overall survival versus HIWI expression in nuclei**. There was no correlation between patients' outcome and the expression level of HIWI in nuclei of tumour cells. *p *values were determined by the log-rank test.

Follow-up information was available for 153 patients for a period of minimum 10 years. The median follow-up time for the 56 patients still alive was 124 months (range 118-155 months) and for the remaining 97 patients died during the follow-up period was 61 months (range 1-139 months). In univariate analysis, patients with low HIWI expression in tumours had a better overall survival than patients with tumour showing moderate or high HIWI expression (*p *< 0.001, *p *= 0.014, respectively), while there was no difference between the moderate expression group and high expression group (*p *= 0.592) (Figure 3). Interestingly, patients with high cytoplasmic HIWI expression tumours had a poorer outcome than that with low cytoplasmic expression tumours (*p *< 0.001) (Figure 4). But we did not see any correlation between patients' outcome and the expression level of HIWI in the nuclei of tumour cells (*p *= 0.979) (Figure 5).

In multivariate Cox's regression analysis, we detected a 2.247-fold increased risk of tumour-related death for patients (*p *= 0.001) whose tumours showed high cytoplasmic HIWI expression compared to patients with low cytoplasmic HIWI expression (Table 3). Still, there was no significant difference in the risk of tumour-related death between patients with high nuclear HIWI expression and low nuclear HIWI expression (*p *= 0.397). Thus, HIWI expression in cytoplasm could be an independent prognostic factor of survival.

**Table 3:** Multivariate analysis of death events

Parameters	Ratio of risk (RR)	95% CI	*P*
Age	1.057	0.806-1.385	0.689
Gender	1.119	0.709-1.767	0.628
Location	1.170	0.785-1.745	0.441
Size	0.925	0.585-1.462	0.739
Lymph node metastasis	2.280	1.429-3.636	0.001*
T stage	1.885	1.204-2.951	0.006*
Histological grade	3.085	2.242-4.247	<0.001*
HIWI in cytoplasm	2.247	1.375-3.670	0.001*
HIWI in nuclei	1.230	0.761-1.988	0.397

* *P *< 0.05

## Discussion

To date, we are the first to disclose the expression of HIWI in human esophageal squamous cell carcinoma and its relation to prognosis. The overexpression of HIWI was previously detected in seminoma, a type of testicular germ cell tumour[19]. Liu *et al*. [16] detected the expression of HIWI in gastric cancer cell line and gastric cancer tissues, which showed that the percentage of HIWI positive cells was dramatically increased from 10% in normal gastric tissues to 76% in gastric cancers. Moreover, its expression was associated with the proliferation of gastric cancer cells. In soft-tissue sarcomas, an increased expression of HIWI was proved to be correlated to a high risk of tumour related death[21]. Also in pancreas adenocarcinoma, alterations in mRNA expression of HIWI were also shown to be associated with increasing risk of tumour-related death in male patients[20]. All of these findings suggest that HIWI may play an important role in development of various cancers.

Esophageal cancer is one of the most aggressive tumours and the overall prognosis for esophageal cancer patients is poor[24]. One of the reasons for the low survival rate is the tumour's intrinsic resistance to many clinical therapies, especially chemotherapy. Chemotherapy often removes the bulk of a tumour mass without preventing tumour recurrence, suggesting the survival of a subset of cancer stem cells. Recent studies have provided experimental evidence for the concept that human tumour growth and metastasis may depend on a small portion cancer stem cells[3].

It has been proposed that HIWI is a key regulator of stem cell pluripotency by controlling cell self-renewal and differentiation[18]. Phenotypically, human preimplantation embryonic cells resemble cancer cells in many ways, especially in their ability to grow indefinitely. Both types of cells undergo deprogramming to a proliferating state and become immortal, self-renewal, and invasive. It has been reported that HIWI is expressed in human tumours but not in normal somatic tissues, in agreement with the hypothesis that embryonic genes are re-activated in tumour cells.

In our cohort, the expression of HIWI was detected in nuclei or/and cytoplasm in 137 (89.5%) of 153 human esophageal squamous cell carcinoma samples with immunohistochemistry. 86 (56.2%) were strongly positive in cytoplasm, while 49 (32.0%) were strongly positive in nuclei. Only a few reports describing the subcellular location of HIWI protein in cells are available. In the previous studies, HIWI was discovered only in cytoplasm[16,20]. But in our series, HIWI expression was observed in both nuclei and cytoplasm, which is consistent with the subcellular location pattern of Piwi expression in germ cells[7,25,26].

Regarding the clinical outcome, no correlation was observed between the level of HIWI expression in nucleus and clinicopathological features, neither was the impact of HIWI expression in nucleus on tumor-related death. However, the expression level of HIWI in cytoplasm was positively associated with histological grade (*P *= 0.011) and T stage (*P *= 0.035) in our present study. Furthermore, patients with high cytoplasmic HIWI expression tumours showed a poorer survival than that with low cytoplasmic expression tumours (*P *< 0.001). Interestingly, when we took both cytoplasmic and nuclear expression into consideration, only correlation between expression level and T stage (*P *= 0.002) could be observed. Moreover, there was no significant difference between the survival of the moderate expression group and high expression group (*p *= 0.592). Our findings suggest that, although the expression of HIWI could be observed in both nucleus and cytoplasm, only HIWI in cytoplasm plays a role in the development of human esophageal squamous cell carcinoma, because it is significantly associated with poorer survival of the patients. Till now, little is known about the biological functions of HIWI, especially about how it functions in epithelial solid cancer. However, increasing evidence supports the notion that Piwi proteins serve an epigenetic role in germline and stem cell maintenance. Miwi and Ziwi, the murine homologue and zebrafish homologue of Piwi family, localize intensely in perinuclear germline granules[27,28], and in particular, Miwi is a component of the chromatoid body, a dynamic cytoplasmic structure that contacts the nuclear periphery repeatedly[29]. Furthermore, a fraction of Miwi comigrates with polysomes on density gradients, suggestive of a large cytoplasmic piRNA complex[27]. Thus, it is possible that HIWI proteins, similar to Piwi proteins in the cytoplasm, may create complex, which impacts on chromatin structure and function during cell division. Therefore, it merits to further study other HIWI family members in the human esophageal squamous carcinoma, in order to better understand their clinical correlations.

## Conclusion

In summary, we have demonstrated for the first time that the stem cell associated gene, HIWI, is expressed in human esophageal squamous cell carcinoma. The higher level of HIWI protein expression in cytoplasm of the esophageal carcinomas cells is significantly associated with higher histological grade, higher T stage and poorer overall survival. Our results highly indicate the involvement of HIWI in carcinogenesis and development of human esophageal squamous cell cancer.

## Competing interests

The authors declare that they have no competing interests.

## Authors' contributions

WH is responsible for laboratory work, data analysis and paper writing. ZW is responsible for laboratory work and data analysis. QW is responsible for data analysis. QF is responsible for data analysis. CS is responsible for antibody preparation. JW is responsible for sample collection and clinical data analyses. KEG is responsible for data analysis, sample collection and clinical data analyses. JMN is responsible for project design and data analyses. ZS is responsible for project design, project support and manuscript preparation. All authors read and approved the final manuscript.

## Pre-publication history

The pre-publication history for this paper can be accessed here:

http://www.biomedcentral.com/1471-2407/9/426/prepub

## References

[B1] Polyak K Hahn WC Roots and stems: stem cells in cancer Nature medicine 2006 12 3 296 300 10.1038/nm1379 16520777

[B2] Soltysova A Altanerova V Altaner C Cancer stem cells Neoplasma 2005 52 6 435 440 16284686

[B3] Reya T Morrison SJ Clarke MF Stem cells, cancer, and cancer stem cells Nature 2001 414 6859 105 111 10.1038/35102167 11689955

[B4] Singh SK Clarke ID Hide T Cancer stem cells in nervous system tumors Oncogene 2004 23 43 7267 7273 10.1038/sj.onc.1207946 15378086

[B5] Wang JC Dick JE Cancer stem cells: lessons from leukemia Trends in cell biology 2005 15 9 494 501 10.1016/j.tcb.2005.07.004 16084092

[B6] Bohmert K Camus I Bellini C AGO1 defines a novel locus of Arabidopsis controlling leaf development The EMBO journal 1998 17 1 170 180 10.1093/emboj/17.1.170 9427751 PMC1170368

[B7] Cox DN Chao A Lin H Piwi encodes a nucleoplasmic factor whose activity modulates the number and division rate of germline stem cells Development (Cambridge, England) 2000 127 3 503 514 10631171 10.1242/dev.127.3.503

[B8] Kuramochi-Miyagawa S Kimura T Yomogida K Two mouse piwi-related genes: miwi and mili Mechanisms of development 2001 108 1-2 121 133 10.1016/S0925-4773(01)00499-3 11578866

[B9] Moussian B Schoof H Haecker A Role of the ZWILLE gene in the regulation of central shoot meristem cell fate during Arabidopsis embryogenesis The EMBO journal 1998 17 6 1799 1809 10.1093/emboj/17.6.1799 9501101 PMC1170527

[B10] Reinke V Smith HE Nance J A global profile of germline gene expression in C. elegans Molecular cell 2000 6 3 605 616 10.1016/S1097-2765(00)00059-9 11030340

[B11] Seipel K Yanze N Schmid V The germ line and somatic stem cell gene Cniwi in the jellyfish Podocoryne carnea The International journal of developmental biology 2004 48 1 1 7 10.1387/ijdb.15005568 15005568

[B12] Tan CH Lee TC Weeraratne SD Ziwi, the zebrafish homologue of the Drosophila piwi: co-localization with vasa at the embryonic genital ridge and gonad-specific expression in the adults Mechanisms of development 2002 119 Suppl 1 S221 224 10.1016/S0925-4773(03)00120-5 14516689

[B13] Cox DN Chao A Baker J A novel class of evolutionarily conserved genes defined by piwi are essential for stem cell self-renewal Genes & development 1998 12 23 3715 3727 9851978 10.1101/gad.12.23.3715 PMC317255

[B14] Hutvagner G Simard MJ Argonaute proteins: key players in RNA silencing Nature reviews 2008 9 1 22 32 10.1038/nrm2321 18073770

[B15] Lingel A Sattler M Novel modes of protein-RNA recognition in the RNAi pathway Current opinion in structural biology 2005 15 1 107 115 10.1016/j.sbi.2005.01.010 15718141

[B16] Liu X Sun Y Guo J Expression of hiwi gene in human gastric cancer was associated with proliferation of cancer cells International journal of cancer 2006 118 8 1922 1929 10.1002/ijc.21575 16287078

[B17] Seto AG Kingston RE Lau NC The coming of age for Piwi proteins Molecular cell 2007 26 5 603 609 10.1016/j.molcel.2007.05.021 17560367

[B18] Sharma AK Nelson MC Brandt JE Human CD34(+) stem cells express the hiwi gene, a human homologue of the Drosophila gene piwi Blood 2001 97 2 426 434 10.1182/blood.V97.2.426 11154219

[B19] Qiao D Zeeman AM Deng W Molecular characterization of hiwi, a human member of the piwi gene family whose overexpression is correlated to seminomas Oncogene 2002 21 25 3988 3999 10.1038/sj.onc.1205505 12037681

[B20] Grochola LF Greither T Taubert H The stem cell-associated Hiwi gene in human adenocarcinoma of the pancreas: expression and risk of tumour-related death British journal of cancer 2008 99 7 1083 1088 10.1038/sj.bjc.6604653 18781170 PMC2567072

[B21] Taubert H Greither T Kaushal D Expression of the stem cell self-renewal gene Hiwi and risk of tumour-related death in patients with soft-tissue sarcoma Oncogene 2007 26 7 1098 1100 10.1038/sj.onc.1209880 16953229

[B22] Parkin DM Bray F Ferlay J Global cancer statistics, 2002 CA: a cancer journal for clinicians 2005 55 2 74 108 10.3322/canjclin.55.2.74 15761078

[B23] Yang H Berner A Mei Q Cytologic screening for esophageal cancer in a high-risk population in Anyang County, China Acta cytologica 2002 46 3 445 452 12040635 10.1159/000326859

[B24] Lin YC Wu MY Li DR Prognostic and clinicopathological features of E-cadherin, alpha-catenin, beta-catenin, gamma-catenin and cyclin D1 expression in human esophageal squamous cell carcinoma World J Gastroenterol 2004 10 22 3235 3239 15484291 10.3748/wjg.v10.i22.3235 PMC4572286

[B25] Megosh HB Cox DN Campbell C The role of PIWI and the miRNA machinery in Drosophila germline determination Curr Biol 2006 16 19 1884 1894 10.1016/j.cub.2006.08.051 16949822

[B26] Saito K Nishida KM Mori T Specific association of Piwi with rasiRNAs derived from retrotransposon and heterochromatic regions in the Drosophila genome Genes & development 2006 20 16 2214 2222 16882972 10.1101/gad.1454806 PMC1553205

[B27] Grivna ST Pyhtila B Lin H MIWI associates with translational machinery and PIWI-interacting RNAs (piRNAs) in regulating spermatogenesis Proceedings of the National Academy of Sciences of the United States of America 2006 103 36 13415 13420 10.1073/pnas.0605506103 16938833 PMC1569178

[B28] Houwing S Kamminga LM Berezikov E A role for Piwi and piRNAs in germ cell maintenance and transposon silencing in Zebrafish Cell 2007 129 1 69 82 10.1016/j.cell.2007.03.026 17418787

[B29] Kotaja N Sassone-Corsi P The chromatoid body: a germ-cell-specific RNA-processing centre Nature reviews 2007 8 1 85 90 10.1038/nrm2081 17183363

